# Some Verifications

**Published:** 1890-12

**Authors:** Ella M. Tuttle

**Affiliations:** New Berlin, N. Y.


### SOME VERIFICATIONS.

Ella M. Tuttle, M. D., New Berlin, N. Y.

Diarrhœa.—Mrs. L. came to me August 11th, complaining of the following symptoms:

A diarrhœa that had lasted her for over a week. She had cured (?) it once by taking black pepper, but it had come on again. Stool scanty, like *cream,* with constant urging. Said she felt as if she could go to stool any time. Much flatus passed with the stool. Colicky pains in the abdomen during and after stool. Weakness of the sphincter ani so that a little of the stool passed involuntarily. She had been troubled with constipation for the last six months and had been liberally dosed by an allopathic physician.

Considering this and the symptom of constant urging, I gave *Nux-vom.*$^{3}$, a dose after every stool. Four doses entirely stopped the diarrhœa.

Is this creamy stool common to Nux? I do not find it in any of the *Materia Medicas.*

Diarrhœa.—Was called July 27th to see Mrs. E., a lady over eighty years old. Found her suffering from much colicky pain in the abdomen, frequent stools of yellow fecal matter streaked with blood. There was soreness over the pit of the stomach and abdomen and some fever. She had been constipated and had taken two pills and eaten some string beans the day before the attack came on. From this fact and the absence of definite symptoms I gave *Nux-vom.*$^{30}$.

July 28th.—Stool yellow and slimy; no blood had passed *involuntarily during her sleep.* I asked her about the pain and soreness in the abdomen and she said "Oh! I don't feel that as long as I keep still, but if I move I feel so bad." Here were my key-notes—stool passed involuntarily during sleep and aggravation from motion. I gave *Bry.*$^3$. She did not have another stool for three days (I kept her on *Sac-lac.* during the time), and then she had a natural fecal movement. The lesson that I drew from this case was that, though *Nux-vom.* is the remedy when the patient has been drugged, yet it will not cure unless the symptoms call for it.

